# Psychiatric Problems in Children and Adolescents with Sickle Cell Disease, Based on Parent and Teacher Reports

**DOI:** 10.5505/tjh.2012.15986

**Published:** 2012-10-05

**Authors:** Özalp Ekinci, Tanju Çelik, Şule Ünal, Cahit Özer

**Affiliations:** 1 Ozalp Ekinci Child and Adolescent Psychiatry Clinic, Hatay, Turkey; 2 Mustafa Kemal University, School of Medicine, Department of Pediatrics, Hatay, Turkey; 3 Antakya State Hospital, Pediatric Hematology Clinic, Hatay, Turkey; 4 Mustafa Kemal University, School of Medicine, Department of Family Medicine, Hatay, Turkey

**Keywords:** Sickle cell disease, psychiatric problems, children, adolescents

## Abstract

**Objective:** This study aimed to investigate the occurrence of psychiatric problems in children and adolescents withsickle cell disease (SCD).

**Material and Methods:** The Child Behavior Checklist for ages 4-18 years (CBCL/4-18), Conners’ Parent RatingScale (CPRS), Conners’ Teacher Rating Scale (CTRS-R), and The Turgay DSM-IV Based Child and Adolescent BehaviorDisorders Screening and Rating Scale, clinician and parent forms (T-DSM-IV-S) were given to the caregivers and teachersof 31 children with SCD aged between 7-18 years and the caregivers and teachers of 34 age matched controls with irondeficiencyanemia.

**Results:** The SCD patients had higher scores on all 4 of scales. Among the subscales, internalizing problems, andattention problems were more prominent in the SCD patients.

**Conclusion:** Children and adolescents with SCD appear to have an increased risk for psychiatric problems. Regularpsychological evaluation and referral to child and adolescent psychiatry clinics may facilitate timely diagnosis andeffective treatment of at-risk children and adolescents.

## Abstract (tr)

**Amaç:** Bu çalışma Orak Hücre Anemisi (OHA) tanılı çocuk ve ergenlerde psikiyatrik sorunların varlığını incelemeyi amaçlamıştır. Gereç ve Yöntemler: 4-18 yaş arası için Çocuk Davranış Kontrol Listesi (ÇDKL/4-18), Conners Ebeveyn Derecelendirme Ölçeği (CEDÖ), Conners Öğretmen Derecelendirme Ölçeği (CÖDÖ-R) ve Turgay DSM-IV’e dayalı Çocuk ve Ergen Davranış Bozuklukları Tarama ve Derecelendirme Ölçeği, klinisyen ve ebeveyn formları (T-DSM-IV-Ö) 7-18 yaş arası OHA tanılı 31 çocuğun ve 34 yaş olarak eşlenmiş demir eksikliği anemisi tanılı kontrol grubunun bakım vericileri ve öğretmenlerine verildi.

**Bulgular:** OHA tanılı çocuk ve ergenler her 4 ölçekte de daha yüksek puanlar aldılar. Ölçek alt puanlarında içe atım sorunları ve dikkat sorunları öne çıkıyordu.

**Sonuç:** OHA tanılı çocuk ve ergenlerin psikiyatrik sorunlar açısından yüksek riskte olduğu görünmektedir. Olgulara düzenli psikolojik değerlendirme yapılması ve çocuk ve ergen psikiyatri kliniklerine refere edilmesi risk altında olan olguların zamanında tanısı ve etkin tedavisine katkı sağlayabilir.

## INTRODUCTION

Sickle cell disease (SCD) is a genetic hemoglobin disorder characterized by a chronic course and disabling complications. SCD patients experience episodic complications affecting multiple organ systems and an increased risk of infections. Recurrent, acute pain that often requires emergency management is the hallmark of SCD and negatively affects quality of life. Children and adolescents with SCD face many challenges associated with living with a chronic condition that requires lifelong medical management. Treatment and hospitalization of SCD patients of school age can further disrupt everyday life due to absence from school [1,2]. As observed in children and adolescents with other chronic illnesses, the current literature suggests that those with SCD may be at risk for adjustment problems and impaired psychosocial functioning [2,3,4]

A number of studies have shown that the frequency of neuropsychiatric problems in children and adolescents with SCD is higher than that in normal controls [5]. Among the neuropsychiatric problems, depression, anxiety disorders, learning problems, and attention deficit are commonly reported [6,7,8]. The present study aimed to investigate the frequency of psychiatric problems, with a special emphasis on attention-deficit/hyperactivity disorder (ADHD) symptoms, in children and adolescents with SCD.

## MATERIALS AND METHODS

**Study group **

The study included SCD patients aged between 7-18 years that presented to the Antakya Thalassemia Center, Hatay, Turkey, between May 2010 and May 2011. The study protocol was approved by the Mustafa Kemal University, School of Medicine Ethics Committee. Prior to administration, the study questionnaires were explained to the participants by a child and adolescent psychiatry specialist, and informed consent was provided by the adolescent patients and the parents’ of pediatric patients. Patients with documented mental retardation (MR) were excluded from the study, as were those diagnosed with MR at the time of the study process. From among the 35 SCD patients that initially gave consent, 31 fulfilled the study requirements and participated in the study. The control group included 34 children and adolescents with iron deficiency anemia that presented to the Antakya Mustafa Kemal University Hospital Pediatric Clinic. 

**Tools**

**Conners’ Parent Rating Scale (CPRS)**

Conners’ Parent Rating Scale (CPRS) is a 48-item Likert- type scale used to assess problematic behaviors in children [9]. In addition to a total score, there are 5 subscale scores: Conduct problems; impulsivity and hyperactivity; learning problems; anxiety; psychosomatic problems. Dereboy et al. reported that the CPRS-Turkish Version is valid and reliable for use in the Turkish population [10]. 

**Conners’ Teacher Rating Scale-Revised (CTRS-R)**

Conners’ Teacher Rating Scale (CTRS-R) is a commonly used teacher rating scale for diagnosing behavioral problems in children [11]. The 28-item CTRS-R provides a total score and 3 subscale scores: Attention deficit; hyperactivity; conduct problems. Şener et al. reported that the CTRS-R-Turkish Version is valid and reliable for use in the Turkish population [12]. 

**The Turgay DSM-IV Based Child and Adolescent Behavior Disorders Screening and Rating Scale, clinician and parent forms (T-DSM-IV-S)**

The Turgay DSM-IV Based Child and Adolescent Behavior Disorders Screening and Rating Scale, clinician and parent forms (T-DSM-IV-S) was developed by Turgay [13]) and consists of 42 items that measure attention-deficit, hyperactivity, impulsivity, and disruptive behaviors. In the present study a shorter version of the scale was used probthat included 9 attention-deficit items, 6 hyperactivity items, and 3 impulsivity items. Ercan et al. reported that the T-DSM-IV-S Scale-Turkish Version is valid and reliable for use in the Turkish population [14]. 

**Child Behavior Checklist for Age 4-18 years (CBCL/4-18)**

The Child Behavior Checklist for age 4-18 years (CBCL/4-18) was developed by Achenbach and Edelbrock to evaluate adequate and problematic behavior in children, based on parental reporting [15]. The 112-item CBCL/4-18 provides a total score, internalizing and externalizing scores, and somatic complaints, anxiety/depression, social problems, thought problems, attention problems, delinquent behaviors, and aggressive behaviors subscale scores. In the present study the items’ T scores were calculated with Cross Informant Program V 4.0 [16] and entered into the study database. Erol and Şimşek reported that the CBCL/4-18-Turkish Version is valid and reliable for use in the Turkish population [17]. 

**Statistical Analysis**

All study data were analyzed using SPSS v.13.0 software. Student’s t test was used to compare patient group and control group means. The level of statistical significance was set at P=0.05.

## RESULTS

Mean age in the SCD group (n=31) was 12.6±3.2 years, versus 12.0±2.9 years in the control group (n=34). In all, 19 (61%) SCD patients and 16 (47%) controls were male. Table 1 shows the CBCL/4-18 total and subscale scores in the 2 groups. Mean CBCL/4-18 total score was significantly higher in the SCD group (P=0.02), as was the internalizing subscale score (P=0.01), withdrawn subscale score (P=0.001), somatic complaints subscale score (P=0.05), anxiety/depressive symptoms subscale score (P=0.03), thought problems subscale score (P=0.01), and attention problems subscale score (P=0.01).

CPRS scores in the SCD and control groups are shown in Table 2. CPRS total score (p=0.001), conduct problems subscale score (P=0.002), anxiety subscale score (P=0.001), and psychosomatic problems subscale score (P=0.001) were significantly higher in the SCD group. CTRSR scores in the SCD and control groups are shown in Table 3. The CTRS-R attention deficit subscale score was significantly higher in the SCD group (P=0.002). T-DSM-IV-S scores in the SCD and control groups are shown in Table 4. T-DSM-IV-S total score (P=0.001), inattentiveness subscale score (P=0.002), and hyperactivity/impulsivity subscale score (P=0.001) were significantly higher in the SCD group. 

## DISCUSSION

Hemoglobinopathies are among the most common genetic diseases in Turkey. The incidence of the SCD trait is 10.0% in the Çukurova region of southern Turkey [18]. In the present study the frequency of psychiatric problems in children and adolescents with SCD treated at a single center in this region was investigated. As compared to the control group, the SCD patients had higher psychiatric problem scores on all 4 psychiatric scales administered. 

The SCD patients had significantly higher CBCL/4-18 subscale scores. Among the CBCL/4-18 subscale scores, internalizing, including somatic complaints, anxiety/ depressive symptoms, and thought problems scores were higher in the SCD group. Internalizing problems and emotional symptoms (that meet or do not meet DSM-IV criteria) commonly occur in children with chronic medical diseases [19]. Previous studies on children and adolescents with SCD reported high frequencies of internalizing problems, including symptoms of depression and anxiety [6,20,21,22]. These symptoms may be attributed to be born with a chronic illness, the unpredictable painful nature of the disease and the feelings of frustration or a sense of injustice that the children have been afflicted [2]. Somatic symptoms are important in the differential diagnosis of children with SCD. Children with SCD may experience chronic pain directly related to SCD or in the form of additional somatic psychiatric symptoms. Somatic complaints may also underlie unrecognized depression or anxiety; therefore, psychiatric consultation should be considered for children and adolescents with SCD that have chronic somatic complaints. 

In the present study the parent-rated CPRS total score and conduct problems subscale, anxiety subscale, and psychosomatic problems subscale scores were higher in the SCD group, whereas the teacher-rated CTRS-R scores showed that the number of children and adolescents with attention deficit was higher in the SCD group. This discrepancy between parent- and teacher-rated scores could have been due to numerous factors. Children with chronic medical diseases like SCD usually experience social withdrawal and isolation at school [4,20,23,24,25]. For instance, children with SCD are usually limited from physical exercise. Frequent hospitalization and absence from school, and related academic failure may also contribute to the social isolation of children and adolescents with SCD [26,27]. As such, SCD patients are usually observed as silent and withdrawn at school [2,4,5,27]. In contrast to school where authority and rules are strict, at home SCD patients may feel free to express their feelings and behavioral characteristics. Parents’ fears about their children with SCD may also lead to a parenting style characterized by low-level authority at home and unresponsiveness to problematic behaviors. Taken together, the excessive control and/or suppressed behavioral problems of children and adolescents with SCD at school may be more apparent at home. 

In the present study the SCD patients had higher T-DSM-IV-S total, inattentiveness subscale, and hyperactivity/ impulsivity subscale scores. CTRS-R and CBCL/4-18 attention-deficit subscale scores in the SCD group were also higher than in the control group. The present findings are in accordance with those of previous studies that reported children with SCD have an increased risk of concentration problems [8,20,28]— especially sustained attention [8]. Although not definitively proven, deficits in attention are often related to the presence and severity of cerebral injury [28,29,30]. Frontal lobe abnormalities, especially anterior infarcts, appear to play a major role in attention deficit [8,31]. Although there is an available literature on attention deficit in SCD, to the best of our knowledge the present study is the first to link SCD with ADHD. It must be noted that the parent-rated CPRS used in the present study does not indicate a clinical diagnosis of ADHD, which must be based on a structured clinical interview; however, the possible comorbidity of ADHD in SCD may not be considered surprising. Available data show that the 2 conditions share some neurocognitive features. Attention deficit, executive functions, and working memory are the well-known basic cognitive components of ADHD [32], and have been shown to be impaired in children with SCD [8,33,34]. 

As the present study used a cross-sectional design, the direct causal relationship of the variables could not be evaluated. The high frequency of psychiatric problems in the SCD group may have, at least partly, been due to some endogenous factors, including personality characteristics. Investigation of personality characteristics in SCD and control groups in a prospective study might yield interesting findings. In addition, whether a high frequency of psychiatric problems is specific to SCD or is also valid for other hemoglobinopathies remains unknown. Multicenter studies with larger patient groups, including children with other hemoglobinopathies, are necessary to more fully elucidate the relationship between SCD and psychiatric problems in children and adolescents. 

The present study has several limitations, including a small sample size, use of subjective questionnaires for data collection, and lack of a structured psychiatric interview. In addition, the prevalence of silent cerebral infarcts was not examined in the present study, which is also considered a limitation. As such, the extent to which the presence of cerebral infarcts was responsible for the frequency and types of psychiatric problems observed in the present study is unknown. In contrast, the major strength of the present study is its use of both parent- and teacher-rated data. Previously, most relevant studies used only parent-rated data. As school is a major domain in a child’s life, teachers’ objective information may be considered valuable.

## CONCLUSION

Children and adolescents with SCD appear to be at increased risk for psychiatric problems. According to the present findings, internalizing symptoms and attention problems were the most common psychiatric symptoms in the SCD patients. Thus, early identification and frequent monitoring of psychiatric problems in children with SCD is critical. Regular psychological evaluation and referral of at-risk children and adolescents to child and adolescent psychiatry clinics may facilitate timely diagnosis and effective treatment.

**Conflict of Interest Statement**

The authors of this paper have no conflicts of interest, including specific financial interests, relationships, and/or affiliations relevant to the subject matter or materials included.

## Figures and Tables

**Table 1:**
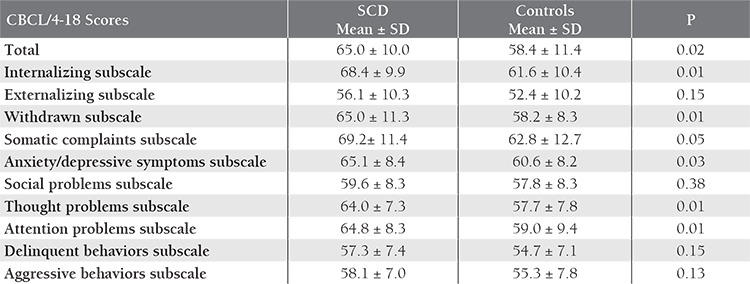
CBCL/4-18 scores in the SCD and control groups

**Table 2:**
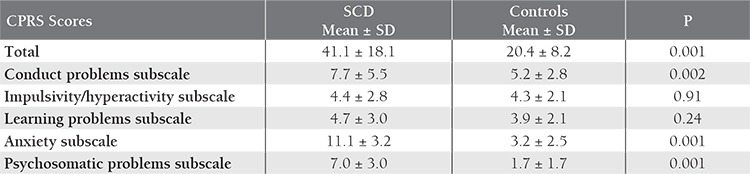
CPRS scores in the SCD and control groups.

**Table 3:**
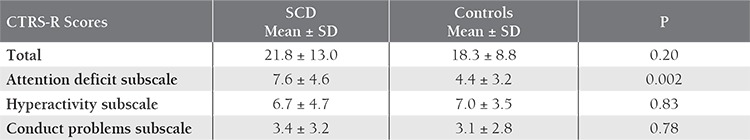
CTRS-R scores in the SCD and control groups

**Table 4:**
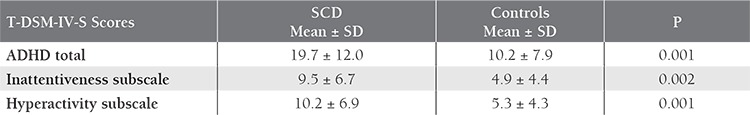
T-DSM-IV-S scores in the SCD and control groups
